# Knowledge, attitudes, and practices towards seasonal influenza vaccination among pregnant women and healthcare workers: A cross‐sectional survey in Afghanistan

**DOI:** 10.1111/irv.13101

**Published:** 2023-03-21

**Authors:** Shahira Shahid, Shafi Kalhoro, Hajra Khwaja, Mohammad Asif Hussainyar, Junaid Mehmood, Muhammad Farrukh Qazi, Abdinasir Abubakar, Shaza Mohamed, Wasiq Khan, Fyezah Jehan, Muhammad Imran Nisar

**Affiliations:** ^1^ Department of Pediatrics and Child Health Aga Khan University Karachi Pakistan; ^2^ Aga Khan University, Academic Projects Afghanistan Kabul Afghanistan; ^3^ WHO Regional Office for the Eastern Mediterranean Cairo Egypt

**Keywords:** attitude, healthcare workers, influenza, knowledge, practice, pregnant women, vaccination

## Abstract

**Background:**

Despite recommendation by the World Health Organization (WHO), influenza vaccination coverage among high‐risk groups remains suboptimal in Afghanistan. This study aims to document the knowledge, attitudes, and practices of seasonal influenza vaccine uptake among two priority groups, pregnant women (PWs) and healthcare workers (HCWs).

**Methods:**

This cross‐sectional study enrolled PWs and HCWs in Kabul, Afghanistan, from September to December 2021. Data on vaccine intention and uptake, knowledge, and attitudes towards vaccination were collected. Simple linear regression was used to predict the impact of sociodemographic characteristics on the KAP score.

**Results:**

A total of 420 PWs were enrolled in Afghanistan. The majority (89%) of these women had never heard of the influenza vaccine but 76% intended to receive the vaccine. Of the 220 HCWs enrolled, 88% were unvaccinated. Accessibility and cost were factors which encouraged vaccination among HCWs. Fear of side effects and affordability were identified as key barriers. HCWs reported high level of vaccine intention (93%). PWs aged under 18 years (*β*: 6.5, *P* = 0.004), between 18 and 24 years (*β*: 2.9, *P* = 0.014), currently employed (*β*: 5.8, *P* = 0.004), and vaccinated against COVID‐19 (*β*: 2.8, *P* = 0.01) were likely to have a higher attitude score. Among HCWs, being female was a predictor for poor vaccination practice (*β*: −1.33, *P* < 0.001) whereas being vaccinated against COVID‐19 was a predictor for higher practice score (*β*: 2.4, *P* < 0.001).

**Conclusion:**

To increase influenza vaccination coverage among priority groups, efforts should be made to address issues such as lack of knowledge, limited availability, and cost barriers.

## 1 INTRODUCTION

Influenza is an infectious disease caused by the influenza virus.^1^ According to the World Health Organization (WHO) estimates, there are one billion influenza cases every year, of which three to five million are of severe illness resulting in 290,000–650,000 deaths globally.^2^ There are certain vulnerable groups, that is, the elderly (>65 years), children younger than 5 years, immunosuppressed patients, healthcare workers (HCWs), and pregnant women (PWs) who experience higher mortality rates and severe cases than healthy adults.^3^ Antiviral drugs can be used to treat influenza; however, vaccination remains the most effective strategy to avert influenza‐related morbidity, mortality, and economic consequences.^2^ Seasonal influenza vaccines are currently available as inactivated and live attenuated types in either trivalent or quadrivalent formulations with efficacy estimates ranging from 16% to 76%.^2^ From 2019 to 2020, vaccination alone prevented about 7.5 million influenza illnesses, 3.7 million medical visits, 105,000 hospitalizations, and 6300 deaths worldwide.^4^, ^5^

Thus, the Strategic Advisory Group of Experts on Immunization (SAGE) by the WHO has recommended seasonal influenza vaccination to protect vulnerable high‐risk groups such as HCWs, PWs, children aged 6 to 59 months, persons with underlying chronic conditions, and the elderly against severe influenza‐associated disease.^6^ Vaccination of HCWs reduces the risk of disease transmission among patients. In addition, vaccination of PWs effectively prevents influenza in both PWs and their infants under 6 months of age, who are ineligible for the vaccine.^6^ Despite these recommendations, Afghanistan lacks a national‐level seasonal influenza vaccination policy and vaccine coverage among the priority groups remains suboptimal. The vaccines are only available in the private sector, and those who wish to receive the vaccine must pay out‐of‐pocket. Additionally, there is a lack of understanding regarding influenza and its prevention, recommendation by the healthcare providers and concerns about the vaccine safety during pregnancy among the population.^7^

This study aims to document the enablers and barriers of seasonal influenza vaccine uptake among two priority groups, PWs and HCWs in Afghanistan.

## 2 METHODS

### 2.1 Study design

We conducted independent community‐based cross‐sectional surveys for PWs and HCWs in Kabul, Afghanistan, from September to December 2021. PWs and HCWs were enrolled from the French Medical Institute for Children (FMIC), the Malalai Maternity Hospital, and Rabia Balkhi Maternity Hospitals in Kabul city and randomly selected clinics in the districts of Bagrami and Deh Sabz, Kabul. A convenience sample was taken where HCWs at the above‐named health facilities and PWs coming for antenatal visits were enrolled after obtaining written informed consent.

### 2.2 Study tools

Quantitative data were collected using separate standardized tools for PWs and HCWs. The tools were adapted from a generic Partnership for Influenza Vaccine Introduction (PIVI) survey tool by researchers at the Aga Khan University, Pakistan, to suit local needs taking inputs from WHO EMRO experts and other research team members. The first section of the questionnaire comprised of 30 and 40 questions for HCWs and PWs, respectively. These questions assessed participants' general knowledge and attitudes regarding influenza and its vaccines. In addition, PWs were asked about their pregnancy status, health history, and antenatal care visits. The response options could be “yes,” “no,” “don't know,” or a set of alternatives/possible answers. Both PWs and HCWs were given 12 statements which assessed attitudes towards the influenza vaccine using a 5‐point Likert scale from *strongly agree* to *strongly disagree*. For each component, higher values corresponded to more positive beliefs and attitudes towards the vaccine and higher levels of knowledge about the vaccination.

The second section asked about sociodemographic characteristics such as gender, age, employment status, and COVID‐19 vaccination status. The English language questionnaire was first translated into a regional language (Dari) and a back translation was done to ensure consistency. The translated tools were pretested in a small sample. The questionnaire took 15–20 min to complete and was administered by trained community health workers (CHWs)/data collectors at all study sites on an online mobile app built on Redcap software. Data were then transferred and stored at a local server at Aga Khan University Hospital daily with restricted access.

### 2.3 Sample size

We assumed an adequate knowledge level of 50% among HCWs and PWs for a maximum sample size with a margin of error of ±5% and an alpha level of 0.05 resulting in a sample size of 220 in HCWs and 420 in PW after considering 10% incomplete responses. The sample size assumptions were varied to account for feasibility given the COVID‐19 and security conditions in Afghanistan.

### 2.4 Statistical analysis

Mean ± SD were reported for continuous variables, median (IQR) were reported for non‐normal discrete variables, and numbers with percentages were reported for categorical variables. Attitude scores were obtained by adding Likert scale points for each question, values ranged from *strongly agree* = 5 to *strongly disagree* = 1. Similarly, knowledge and practice scores were calculated. Response values were set as “yes” = 2, “no” = 1, and “don't know” = 0. Simple linear regression model was used to predict the knowledge, attitude, and practice scores at univariate level using sociodemographic characteristics; all the factors with *P* value < 0.10 were considered for multivariate analysis. A final multivariate model was build using stepwise backward elimination, *P* value < 0.05 was considered statistically significant. The level of significance was set at 0.05. STATA version 17.0 was used for all analyses.

### 2.5 Ethical considerations

Ethical approval was obtained from the Aga Khan University's ethical review committee (ERC 2021‐6649‐18992) and the Institutional Review Board (IRB) in Afghanistan (IRB Code No: A.1121.0384). No personal identifiers were collected. Interviews were conducted in a private and secluded place after obtaining consent and taking care of confidentiality.

## 3 RESULTS

### 3.1 Sociodemographic characteristics of the study participants

Table 1 describes the sociodemographic characteristics of the 420 enrolled PWs. Most respondents (33.5.0%) were aged between 25 and 29 years, 67.1% received no formal education, and only 4.5% were employed. Mean gestational age at the time of interview was 25.3 ± 11.0 weeks. Median number of total pregnancies was 4 (2–5). Only 17.4% of the PW had received the COVID‐19 vaccine.

**TABLE 1:** Sociodemographic characteristics of pregnant women in Afghanistan.

Characteristics	*N* = 420
	*n* (%)
Age, years	*n* = 418
Under 18	15 (3.6%)
18–24	88 (21.1%)
25–29	140 (33.5%)
30–34	108 (25.8%)
35–39	52 (12.4%)
40–49	15 (3.6%)
Highest level of education	*n* = 407
No formal education	273 (67.1%)
Primary	58 (14.3%)
Secondary	48 (11.8%)
Higher secondary or above	28 (6.9%)
Currently employed^a^	19 (4.5%)
Gestational age in weeks at the time of enrolment (mean ± SD)	25.3 ± 11.0
Total number of pregnancies including current, median (IQR)	4 (2–5)
Total number of children, median (IQR)	2 (1–4)
Vaccinated against COVID‐19	73 (17.4%)

^a^ Currently employed: *n* = 418.

Table 2 describes the sociodemographic characteristics and COVID‐19 vaccination status of the 220 HCWs enrolled in the study. Mean age was 35.3 ± 10.7 years. Majority of the HCWs (90.5%) were females, 30.9% were doctors, and 62.7% were employed in the obstetrics/gynecology department. COVID‐19 vaccination rates were high (81.8%) in the cohort.

**TABLE 2:** Sociodemographic characteristics of healthcare workers in Afghanistan.

Characteristics	*N* = 220
	*n* (%)
Age, years (mean ± SD)	35.3 ± 10.7
Gender	
Male	21 (9.5%)
Female	199 (90.5%)
Highest level of education	
Primary	10 (4.5%)
Secondary	0 (0.0%)
Vocational education	61 (27.7%)
Graduation	119 (54.1%)
Postgraduate	30 (13.6%)
Occupation	
Doctor	68 (30.9%)
Nurse	49 (22.3%)
Midwife	96 (43.6%)
Others	7 (3.2%)
Department/unit	
Medicine/ICU	33 (15.0%)
Pediatrics/NICU	24 (10.9%)
Surgery	16 (7.3%)
Obstetrics/gynecology	138 (62.7%)
Others	9 (4.1%)
Years of experience	
<5 years	68 (30.9%)
5–9 years	59 (26.8%)
>9 years	93 (42.3%)
Vaccinated against COVID‐19	
Yes	180 (81.8%)
No	40 (18.2%)

### 3.2 Knowledge, attitudes, and practices of PWs regarding influenza vaccine

**FIGURE 1:**
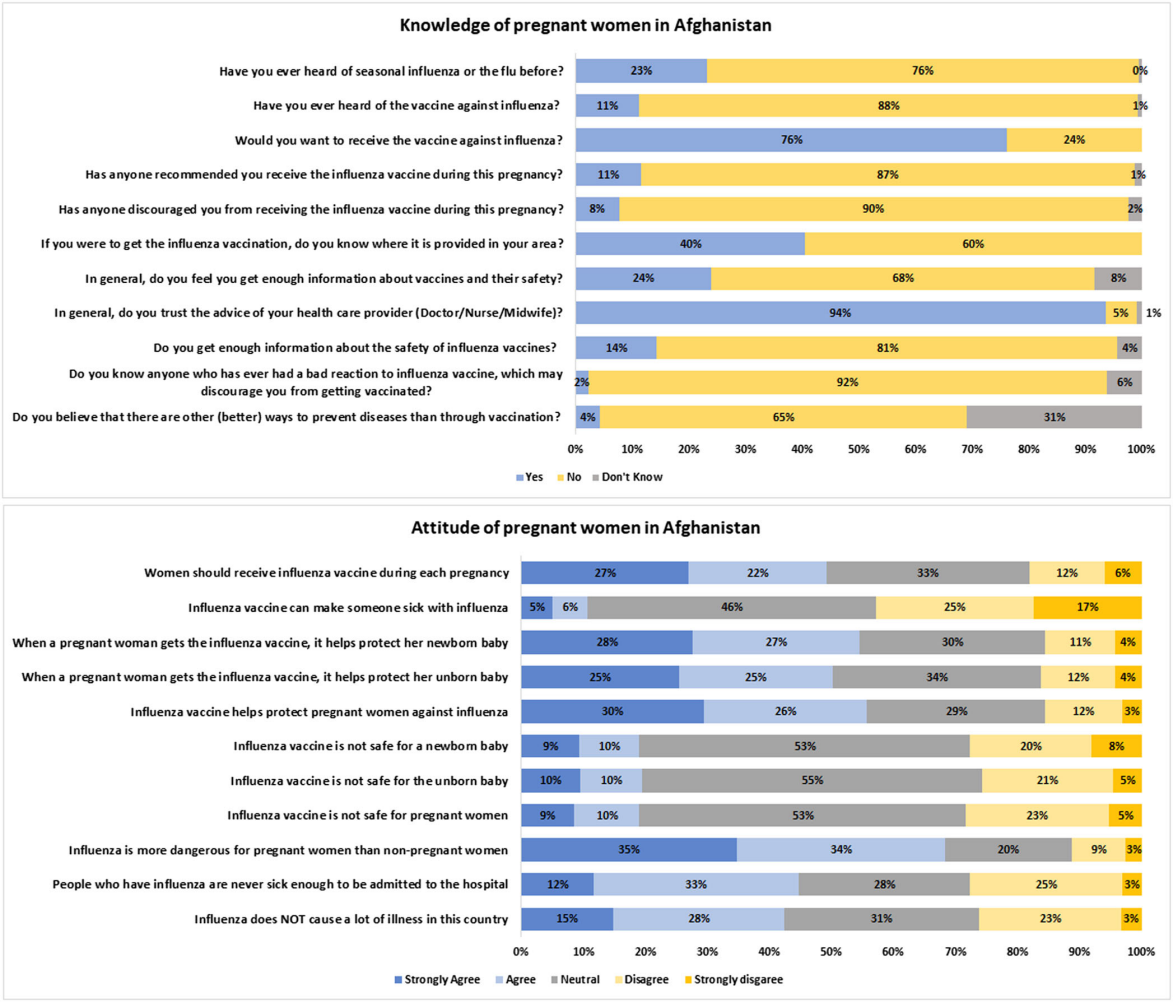
Knowledge and attitudes of pregnant women towards influenza vaccine.

**FIGURE 2:**
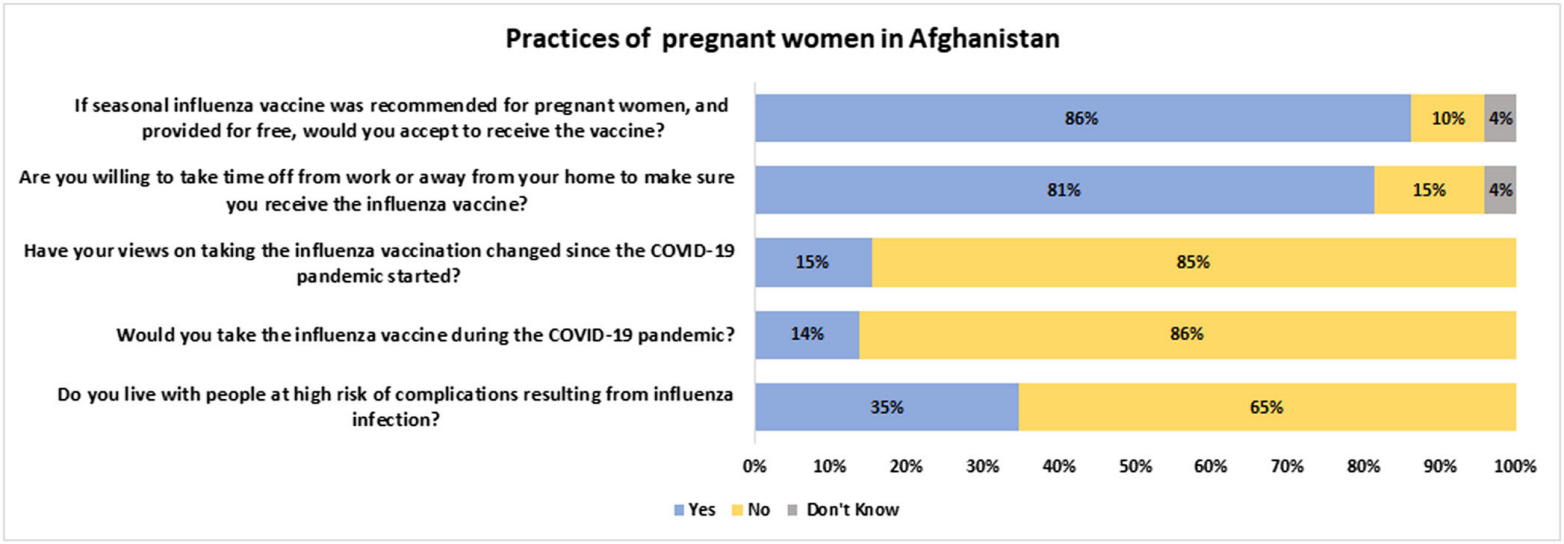
Influenza vaccine intention and uptake among pregnant women.

Figures 1 and 2 describe the knowledge, attitudes, and practices of the PWs. Overall, 11% of the PW had heard of the vaccine previously and were recommended by HCWs to receive the vaccine during their antenatal visit, 76% of the PW wanted to receive the vaccine, 40% knew where it would be available in their area, 8% were discouraged by their community from receiving the vaccine during pregnancy, 94% of the PW trusted their healthcare provider's recommendations, 28% of the PW strongly agreed that the vaccine protected their unborn baby while 9% believed that it was not safe for PWs. Eighty‐six percent of the PWs were willing to get the vaccine if it was recommended to them and available free of cost. However, a similar proportion denied receiving the vaccine during the COVID‐19 pandemic.

### 3.3 Knowledge, attitudes, and practices of HCWs regarding influenza vaccine

**FIGURE 3:**
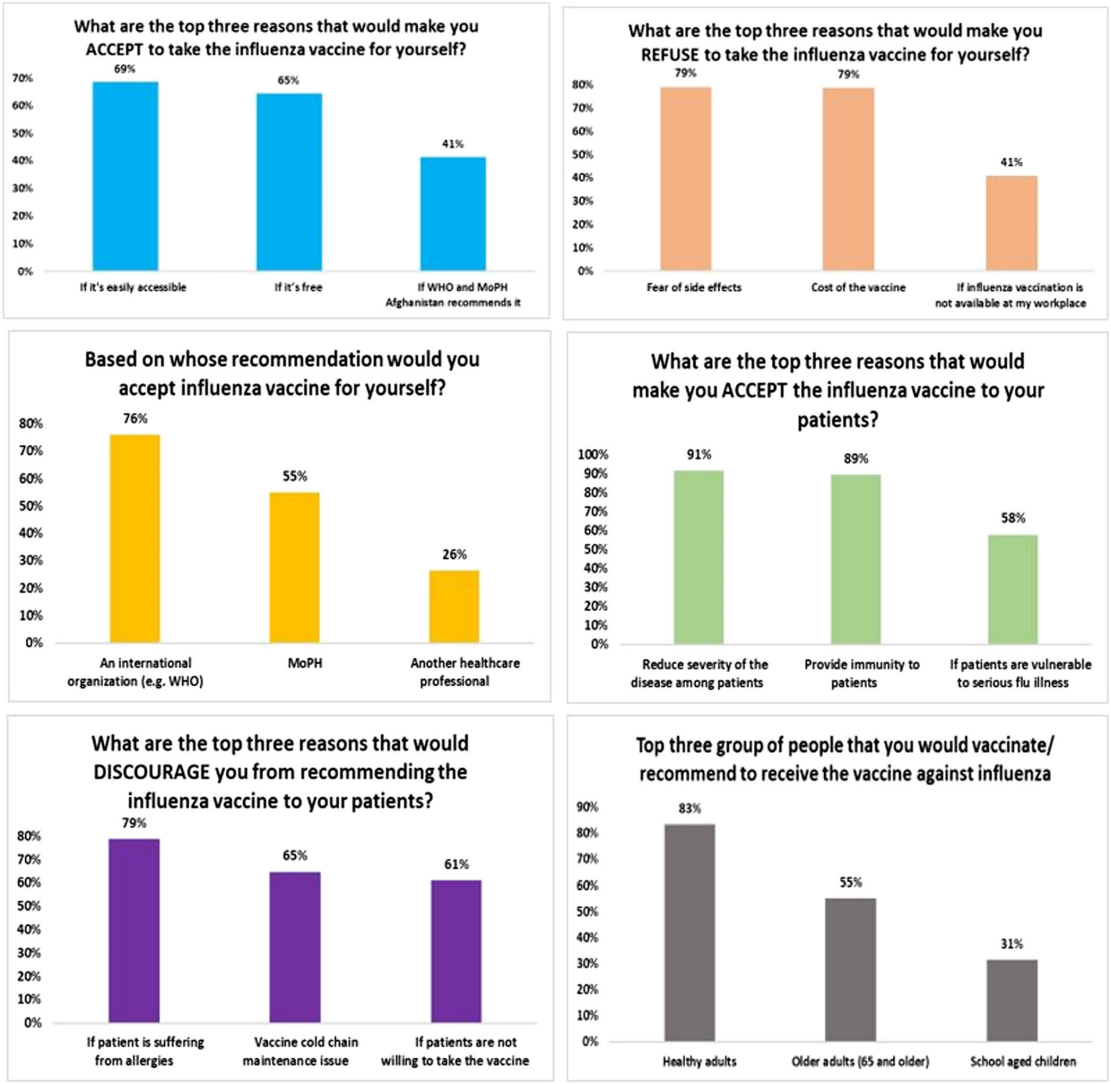
Knowledge of the healthcare workers regarding influenza vaccine.

**FIGURE 4:**
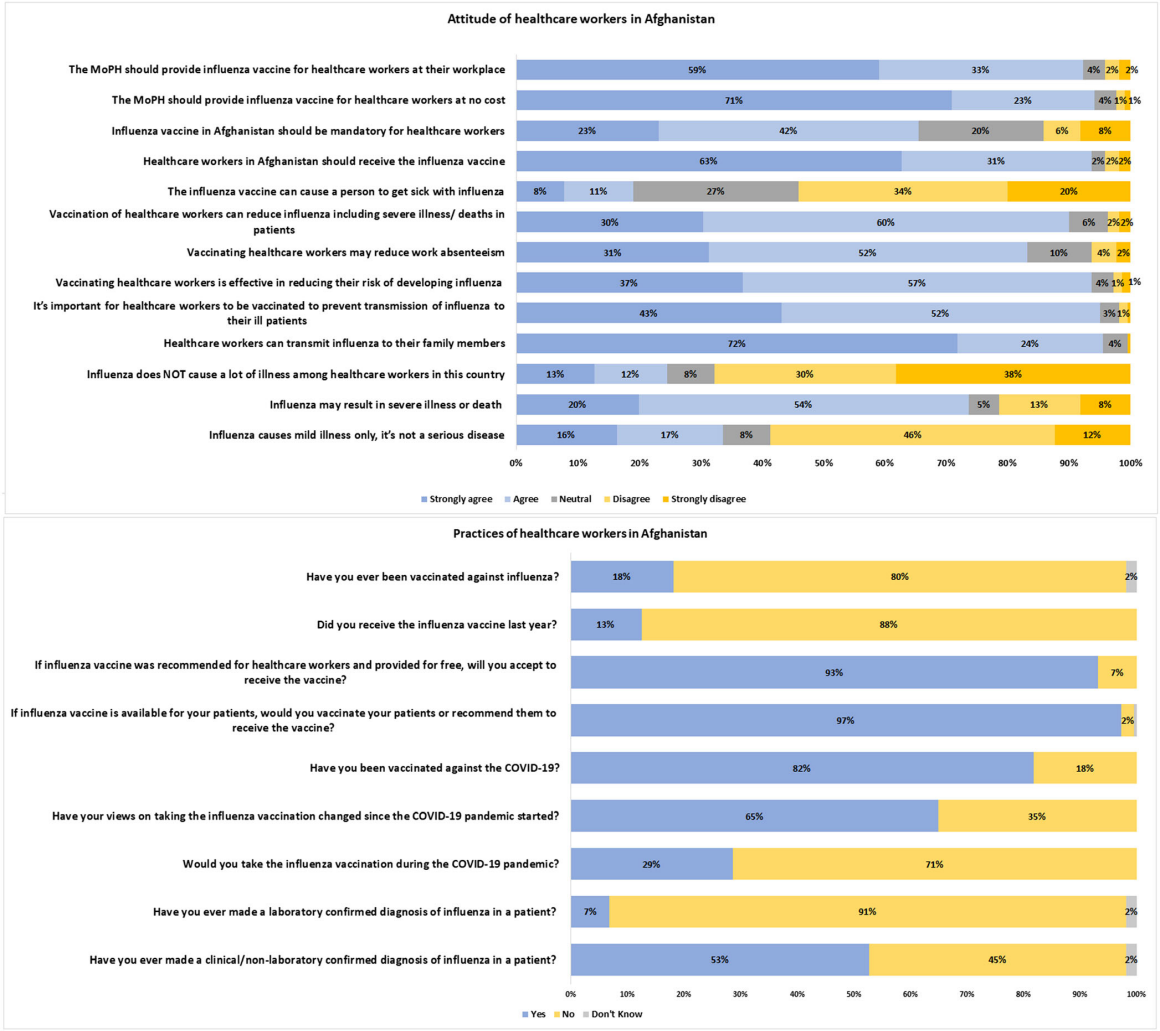
Attitudes and practices of the healthcare workers.

Figure 3 describes the knowledge of influenza vaccine among the 220 HCWs. Majority of the HCWs believed that vaccine reduced disease severity (91%) and provided immunity (89%). According to HCWs, the three most important groups to be vaccinated should be healthy adults (83%), older adults (65 years and above) (55%), and school‐aged children (31%). Most common reasons for vaccine refusal among HCWs were fear of side effects (79%), cost of the vaccine (79%), and unavailability of vaccine (41%) whereas accessibility (69%) and cost (65%) were the most common reasons for vaccine acceptance. More than half the HCWs (78%) were willing to take the vaccine if an international organization (such as the WHO) recommended it. Eighty‐eight percent of the HCWs were actually unvaccinated; however, 93% of them intended to get vaccinated and 97% intended to recommend the vaccine to their patients (Figure 4).

### 3.4 Sociodemographic predictors of KAP scores among PWs and HCWs

Tables 3, 4, S1, and S2 describe the adjusted and unadjusted simple linear regression analysis of sociodemographic variables on KAP scores. PWs under the age of 18 years (*β*: 6.5, *P* = 0.004) and 18–24 years (*β*: 2.9, *P* = 0.014) had higher attitude scores as compared with older women. Similarly, women who were currently employed (*β*: 5.8, *P* = 0.004), or had received the COVID‐19 vaccine (*β*: 2.8, *P* = 0.01) were a predictor for higher attitude scores. An education level of higher secondary or above (*β*: −4.51, *P* = 0.01) was significantly associated with a poor attitude score.

**TABLE 3:** Adjusted coefficients with 95% CIs for KAP scores among pregnant women.

Characteristics	Attitude scores	
	Adjusted	
	Co‐eff (CI)	*P* value
Age, years		
Under 18	6.5 (2.05, 10.95)	0.004
18–24	2.87 (0.59, 5.16)	0.014
25–29	Ref	
30–34	0.21 (−1.9, 2.33)	0.842
35–39	−1.22 (−3.93, 1.48)	0.375
40–49	−1.35 (−5.89, 3.19)	0.56
Highest level of education		
No formal or little education		
Primary	−0.74 (−3.12, 1.64)	0.541
Secondary	0.63 (−1.99, 3.25)	0.637
Higher secondary or above	−4.51 (−7.92, −1.09)	0.01
Employment status		
Currently employed	5.79 (1.87, 9.7)	0.004
Not currently employed	Ref	
Gestational age in weeks at the time of enrollment	−0.07 (−0.15, 0)	0.056
Vaccinated against COVID‐19		
Yes	2.83 (0.64, 5.02)	0.011
No	Ref	

**TABLE 4:** Adjusted coefficients with 95% CIs for KAP scores among healthcare workers.

Characteristics	Practice score	
	Adjusted	
	Co‐eff (CI)	*P* value
Age, years	0.02 (0, 0.03)	0.061
Gender		
Male	Ref	
Female	−1.33 (−1.97, −0.69)	<0.001
Department/unit		
Medicine/ICU	‐	
Pediatrics/NICU	−0.19 (−0.88, 0.5)	0.589
Surgery	0.08 (−0.69, 0.85)	0.839
Obstetrics/gynecology	0.1 (−0.42, 0.62)	0.713
Others	1.51 (0.54, 2.48)	0.002
Vaccinated against COVID‐19		
Yes	2.43 (1.98, 2.88)	<0.001
No	Ref	

Among HCWs, female gender (*β*: −1.33, *P* < 0.001) was a predictor for lower practice score whereas being vaccinated against COVID‐19 was associated with a greater probability for higher practice (*β*: 2.4, *P* < 0.001).

## 4 DISCUSSION

We assessed knowledge, attitudes, and practices related to influenza vaccination uptake among two priority groups, that is, HCWs and PWs in Kabul, Afghanistan. We reported 13% of the surveyed HCWs to have received the influenza during the previous flu season. This coverage is comparable with HCWs from other South Asian countries such as Pakistan, India, and Iran.^8^, ^9^, ^10^ Low vaccination rates may be attributed to poor knowledge as 16% of the HCWs believed that influenza is not a serious disease, only 7% had ever made a laboratory‐confirmed diagnosis of influenza in their practice, and majority believed that healthy adults and the elderly were the most important groups to be vaccinated.

Among HCWs, the most common reasons for refusal were fear of side effects (79%), cost (79%), and unavailability of the vaccine (41%). In addition, some HCWs believed that vaccination can cause a person to get sick with influenza. Majority of the vaccine‐related adverse events are mild and resolve spontaneously. Therefore, it is necessary to educate HCWs about influenza vaccine efficacy and safety to alleviate such concerns and enhance vaccine uptake. In our study, 30% of the HCWs strongly agreed and 60% agreed that vaccination of HCWs can reduce influenza including severe illness/deaths in patients which indicates a higher level of collective responsibility towards protecting the community against the disease. HCWs also recognized their role in promoting vaccination in the community as 97% intended to recommend the vaccine to their patients if it was freely available.

Our study revealed that vaccine accessibility and cost were key drivers for vaccine acceptance. Similarly, a previous study from Qatar showed that free vaccination was associated with significant improvement in vaccine uptake among the HCWs.^11^ Majority of the HCWs were willing to receive the influenza vaccine when recommended by international organizations (such as the WHO) or local government. This suggests an opportunity to conduct national immunization campaigns and establish guidelines to improve vaccine adherence among this group. We report 81.8% of the HCWs to be vaccinated against COVID‐19. Additionally, being vaccinated against COVID‐19 was found to be positively associated with higher vaccination practice scores among HCWs, indicating that the COVID‐19 pandemic might have increased their intention to get vaccinated against influenza as reported by Kong *et al*.^12^

We also assessed the knowledge, attitudes, and practices of 420 PWs from Kabul, Afghanistan. A substantial deficit was seen in the knowledge of the participants regarding the disease and its prevention. Only, 23% of the PWs were aware of seasonal influenza, 11% had ever heard of its vaccine, and 28% strongly believed that the vaccine protected their unborn baby. Although majority (76%) of the PW wanted to receive the vaccine, only 11% had received a recommendation of it from HCWs during their antenatal visits. Healthcare provider recommendation is a crucial factor in a PW's decision to receive vaccine as 94% of the PWs in the study trusted the recommendation of their healthcare providers. Our findings highlight potential opportunities to increase influenza vaccine uptake among PWs by sensitizing HCWs regarding the benefits and safety of vaccination during pregnancy. Cost and accessibility were other key barriers identified, if seasonal vaccine was recommended for PW and available free of cost, 86% intended to receive it. A previous survey in India also found that the women were willing to be vaccinated if it were recommended by a healthcare provider and if they were informed of vaccine safety during pregnancy.^13^ In our study, being currently employed was a predictor for higher attitude scores. This may be due to greater autonomy in making personal decisions.

To the best of our knowledge, this is the first KAP study of seasonal influenza vaccination among two priority groups, HCWs and PWs in Afghanistan. We used a standardized tool for data collection and enrolled participants from both public and private healthcare facilities. One of the limitations of our study was that the responses could be subject to recall bias. The participants were conveniently selected from hospitals in Kabul and clinics in the adjoining districts and therefore might not be representative of the whole country. In addition, our study was conducted during the waxing and waning COVID‐19 waves, and thus, we could not achieve a higher sample size.

## 5 CONCLUSION

In conclusion, this study reveals substantial gaps in the knowledge, attitudes, and practices of both HCWs and PWs regarding influenza vaccine which need to be systematically addressed. This study can provide a framework upon which targeted interventions can be developed.

## AUTHOR CONTRIBUTIONS

**Shahira Shahid:** Formal analysis; visualization; writing—original draft; writing—review and editing. **Shafi Kalhoro:** Investigation; methodology; project administration; supervision. **Hajra Khwaja:** Formal analysis; visualization; writing—original draft; writing—review and editing. **Mohammad Asif Hussainyar:** Investigation; methodology; project administration; resources; supervision; writing—review and editing. **Junaid Mehmood:** Data curation; investigation; software; supervision; writing—review and editing. **Muhammad Farrukh Qazi:** Formal analysis; software; visualization; writing—original draft; writing—review and editing. **Abdinasir Abubakar:** Conceptualization; funding acquisition; methodology; project administration; supervision; writing—review and editing. **Shaza Mohamed:** Conceptualization; funding acquisition; methodology; project administration; supervision; writing—review and editing. **Wasiq Khan:** Conceptualization; funding acquisition; methodology; project administration; supervision; writing—original draft; writing—review and editing. **Fyezah Jehan:** Conceptualization; funding acquisition; methodology; writing—original draft; writing—review and editing. **Muhammad Imran Nisar:** Conceptualization; formal analysis; funding acquisition; investigation; methodology; project administration; resources; supervision; visualization; writing—original draft; writing—review and editing.

## CONFLICT OF INTEREST

None declared.

## ETHICS STATEMENT

Ethical approval was obtained from Aga Khan University's ethical review committee (ERC 2021‐6649‐18992) and the Institutional Review Board (IRB) in Afghanistan (IRB Code No: A.1121.0384).

## PATIENT CONSENT STATEMENT

Interviews were conducted after obtaining consent from the study participants and taking care of confidentiality.

### PEER REVIEW

The peer review history for this article is available at https://publons.com/publon/10.1111/irv.13101.

## Supporting information


**Table S1.** Unadjusted co‐efficient with 95% CI for KAP scores among pregnant women
**Table S2.** Unadjusted co‐efficient with 95% CI for KAP scores among healthcare workers

## Data Availability

Data will be available from the corresponding author upon request.
