# Supplementary material for: Knowledge, attitudes, and practices towards seasonal influenza vaccination among pregnant women and healthcare workers: A cross‐sectional survey in Afghanistan
Source: Influenza Other Respir Viruses. 2023 Mar 21;17(3):e13101. doi: 10.1111/irv.13101 (PMC10030354; doi:10.1111/irv.13101)
Supplement: Supplementary file 1 — Table S1. Unadjusted co‐efficient with 95% CI for KAP scores among pregnant women Table S2. Unadjusted co‐efficient with 95% CI for KAP scores among healthcare workers [file IRV-17-e13101-s001.docx]

**Supplementary table S1: Unadjusted co-efficient with 95% CI for KAP scores among pregnant women**

| **Characteristics** | **Knowledge Scores** | | **Attitude Scores** | | **Practice Scores** | |
| --- | --- | --- | --- | --- | --- | --- |
|  | **Unadjusted** | | **Unadjusted** | | **Unadjusted** | |
|  | **Co-eff(CI)** | **P-value** | **Co-eff(CI)** | **P-value** | **Co-eff(CI)** | **P-value** |
| **Age, years** |  |  |  |  |  |  |
| Under 18 | 0.83(-0.11,1.76) | 0.082 | 7.29(2.87,11.7) | 0.001 | 0.51(-0.1,1.11) | 0.1 |
| 18-24 | 0.17(-0.3,0.64) | 0.471 | 2.7(0.49,4.91) | 0.017 | 0.26(-0.05,0.56) | 0.096 |
| 25-29 | Ref |  | Ref |  | Ref |  |
| 30-34 | -0.06(-0.5,0.38) | 0.794 | 0.69(-1.39,2.77) | 0.515 | 0.09(-0.19,0.38) | 0.533 |
| 35-39 | -0.41(-0.97,0.15) | 0.154 | -0.73(-3.36,1.91) | 0.589 | -0.17(-0.53,0.2) | 0.368 |
| 40-49 | -0.57(-1.51,0.36) | 0.23 | -1.31(-5.73,3.1) | 0.559 | -0.49(-1.1,0.11) | 0.11 |
| **Highest level of education** |  |  |  |  |  |  |
| No formal or little education | Ref |  | Ref |  | Ref |  |
| Primary | -0.25(-0.74,0.24) | 0.32 | -0.55(-2.95,1.85) | 0.651 | 0(-0.32,0.32) | 0.991 |
| Secondary | 1(0.47,1.53) | 0 | 2.23(-0.37,4.83) | 0.092 | 0.23(-0.12,0.58) | 0.191 |
| Higher secondary or above | 0.71(0.04,1.3) | 0.038 | -1.74(-5.03,1.55) | 0.3 | 0.04(-0.4,0.48) | 0.867 |
| **Employment status** |  |  |  |  |  |  |
| Currently employed | 0.74(-0.06,1.54) | 0.07 | 5.54(1.7,9.38) | 0.005 | 0.16(-0.37,0.69) | 0.558 |
| Not currently employed | Ref |  | Ref |  | Ref |  |
| **Gestational age in weeks at the time of enrolment** | 0(-0.02,0.01) | 0.79 | -0.08(-0.16,-0.01) | 0.024 | -0.01(-0.02,0) | 0.007 |
| **Total number of pregnancies including this one** | -0.02(-0.09,0.05) | 0.588 | 0.15(-0.2,0.5) | 0.389 | 0.01(-0.03,0.06) | 0.571 |
| **Total number of children** | -0.05(-0.13,0.03) | 0.19 | 0.3(-0.09,0.68) | 0.128 | 0.02(-0.04,0.07) | 0.535 |
| **Vaccinated against Covid-19** |  |  |  |  |  |  |
| Yes | -0.13(-0.57,0.32) | 0.57 | 2.7(0.59,4.8) | 0.012 | 0.21(-0.08,0.5) | 0.16 |
| No | Ref |  | Ref |  | Ref |  |

**Supplementary table S2 Unadjusted co-efficient with 95% CI for KAP scores among healthcare workers**

| **Characteristics** | **Attitude Scores** | | | **Practice Scores** | | |
| --- | --- | --- | --- | --- | --- | --- |
|  | **Unadjusted** | | | **Unadjusted** | | |
|  | **Co-eff(CI)** | **P-value** | **Co-eff(CI)** | | **P-value** |  |
| **Age, years** | 0.04(-0.07,0.15) | 0.488 | 0.03(0.01,0.05) | | 0.002 |  |
| **Gender** |  |  |  | |  |  |
| Male | Ref |  | Ref | |  |  |
| Female | -2.78(-6.69,1.13) | 0.162 | -1.48(-2.22,-0.73) | | <0.001 |  |
| **Highest level of education** |  |  |  | |  |  |
| Primary | Ref |  | Ref | |  |  |
| Secondary | - |  | - | |  |  |
| Vocational education | 2.66(-3.14,8.47) | 0.367 | 0.98(-0.17,2.13) | | 0.095 |  |
| Graduation | -0.1(-5.7,5.51) | 0.973 | 0.96(-0.15,2.07) | | 0.089 |  |
| Postgraduate | 0.77(-5.45,6.98) | 0.808 | 1.03(-0.2,2.26) | | 0.099 |  |
| **Occupation** |  |  |  | |  |  |
| Doctor | Ref |  | Ref | |  |  |
| Nurse | 1.82(-1.29,4.93) | 0.25 | 0.52(-0.09,1.13) | | 0.094 |  |
| Midwife | -3.37(-6,-0.74) | 0.012 | -0.31(-0.83,0.2) | | 0.231 |  |
| Others | 2.33(-4.26,8.93) | 0.487 | 1.85(0.55,3.14) | | 0.005 |  |
| **Department/ Unit** |  |  |  | |  |  |
| Medicine/ICU | Ref |  | Ref | |  |  |
| Pediatrics/NICU | -0.69(-5.27,3.89) | 0.767 | -0.44(-1.32,0.44) | | 0.325 |  |
| Surgery | 2.6(-2.6,7.81) | 0.325 | 0.08(-0.91,1.08) | | 0.872 |  |
| Obstetrics/Gynecology | -1.47(-4.78,1.84) | 0.383 | -0.48(-1.12,0.15) | | 0.134 |  |
| Others | -1.83(-8.25,4.59) | 0.575 | 1.73(0.5,2.96) | | 0.006 |  |
| **Years of experience** |  |  |  | |  |  |
| < 5 years | Ref |  | Ref | |  |  |
| 5-9 years | 1.16(-1.87,4.2) | 0.45 | 0.2(-0.39,0.8) | | 0.502 |  |
| >9 years | 2.1(-0.62,4.82) | 0.13 | 0.38(-0.16,0.91) | | 0.168 |  |
| **Vaccinated against COVID-19** |  |  |  | |  |  |
| Yes | 2.13(-0.85,5.1) | 0.16 | 2.64(2.16,3.11) | | <0.001 |  |
| No | Ref |  | Ref | |  |  |
